# The properties of spontaneous mutations in the opportunistic pathogen *Pseudomonas aeruginosa*

**DOI:** 10.1186/s12864-015-2244-3

**Published:** 2016-01-05

**Authors:** Jeremy R. Dettman, Jacqueline L. Sztepanacz, Rees Kassen

**Affiliations:** Department of Biology and Centre for Advanced Research in Environmental Genomics, University of Ottawa, Ottawa, ON K1N 6N5 Canada; School of Biological Sciences, University of Queensland, Brisbane, QLD 4072 Australia

**Keywords:** Mutation accumulation, Genomics, Mutation bias, Mismatch repair

## Abstract

**Background:**

Natural genetic variation ultimately arises from the process of mutation. Knowledge of how the raw material for evolution is produced is necessary for a full understanding of several fundamental evolutionary concepts. We performed a mutation accumulation experiment with wild-type and mismatch-repair deficient, mutator lines of the pathogenic bacterium *Pseudomonas aeruginosa,* and used whole-genome sequencing to reveal the genome-wide rate, spectrum, distribution, leading/lagging bias, and context-dependency of spontaneous mutations.

**Results:**

Wild-type base-pair mutation and indel rates were ~10^−10^ and ~10^−11^ per nucleotide per generation, respectively, and deficiencies in the mismatch-repair system caused rates to increase by over two orders of magnitude. A universal bias towards AT was observed in wild-type lines, but was reversed in mutator lines to a bias towards GC. Biases for which types of mutations occurred during replication of the leading versus lagging strand were detected reciprocally in both replichores. The distribution of mutations along the chromosome was non-random, with peaks near the terminus of replication and at positions intermediate to the replication origin and terminus. A similar distribution bias was observed along the chromosome in natural populations of *P. aeruginosa*. Site-specific mutation rates were higher when the focal nucleotide was immediately flanked by C:G pairings.

**Conclusions:**

Whole-genome sequencing of mutation accumulation lines allowed the comprehensive identification of mutations and revealed what factors of molecular and genomic architecture affect the mutational process. Our study provides a more complete view of how several mechanisms of mutation, mutation repair, and bias act simultaneously to produce the raw material for evolution.

**Electronic supplementary material:**

The online version of this article (doi:10.1186/s12864-015-2244-3) contains supplementary material, which is available to authorized users.

## Background

Of the many possible genetic routes to adaptation available to a population, the one most often followed depends both on the process of mutation, which creates the raw material for adaptive evolution, and the fitness effects of the resulting mutations. Natural selection affects which mutations are maintained or purged from the population, but all adaptive mutations are drawn from the original pool of raw mutations. A growing body of research on the distribution of fitness effects among beneficial mutations suggests that most have small effects and a few have much larger effects on fitness [1, 2]. By contrast, we know less about the mutational factors that influence the evolutionary potential of a population and species, such as the rate at which mutations arise, the relative frequencies of different types of mutations, and how variable the mutational process is across the genome [3–5]. Our ability to make predictions about the outcome of adaptive evolution at the genomic level, and the probability of parallel evolution in independently evolving populations, relies on a detailed understanding of the mutational process.

To help fill this gap, we have investigated the genetic changes introduced by mutation in wild-type and mutator lines of the opportunistic pathogen, *Pseudomonas aeruginosa*, and ask to what extent mutational variation allows us to predict extant patterns of polymorphism in natural isolates of this species. *P. aeruginosa* is the predominant bacterial species isolated from the respiratory tracts of adult patients with cystic fibrosis (CF) and chronic endobronchial infections by this pathogen are associated with increased morbidity and mortality [6, 7]. There is now abundant evidence that *P. aeruginosa* populations undergo extensive adaptive evolution during the transition from free-living, environmental strains to chronic infections of the CF lung [8, 9]. Moreover, mutator strains, which are characterized by higher mutation rates than wild type, are commonly isolated from CF infections [10]. Such hyper-mutability may facilitate adaptation, in part, by increasing the rate at which beneficial mutations arise [11], especially with respect to several virulence-related phenotypes [12–15]. Given the potential advantages conferred by hyper-mutability, there is interest in comparing how the underlying mutational spectra differ in wild-type and mutator strains.

Our approach uses mutation accumulation (MA) experiments, in which the products of spontaneous mutation arise in a neutral context and are documented prior to the filtering imposed by selection. The MA method involves propagating multiple populations through repeated bottlenecks of a single, randomly chosen individual, thereby greatly reducing the effectiveness of selection. Under these conditions, the fates of all mutations are determined stochastically by genetic drift and mutations are free to accumulate independently of their fitness effects (with the exception of lethals). Whole-genome sequencing (WGS) of MA lines provides a comprehensive catalogue of mutational events that occurred over the time frame of the experiment. Analyzing mutations at the whole-genome level allows for averaging across the local biases present at the individual gene level. The MA approach does not require making the assumption, common to more comparative approaches that use DNA sequence variation from natural populations, that particular sites such as synonymous codon positions are, in fact, neutral with respect to fitness. Evidence from both comparative and experimental studies suggests that this is not always the case [16–18], reinforcing the potential value of MA experiments for gaining insight into the mutational process.

Here we determine the rate, spectrum, distribution, leading/lagging bias, and context-dependency of spontaneous mutations by sequencing the genomes of 36 *P. aeruginosa* lines after ~2500 generations of mutation accumulation. When possible, we use these data to investigate the contribution of mutation to extant levels of polymorphism among clinical *P. aeruginosa* isolates. Comparisons between wild-type and mutator lines are made to reveal what types of pre-mutation errors are most commonly made during the DNA replication process, and which pre-mutations are preferentially fixed by a functional mismatch-repair (MMR) system. We also compare the nature of the mutational patterns we observe in *P. aeruginosa* with those from other studies that have applied the MA-WGS approach.

## Results

### Genome-wide mutation rates

**Fig. 1:**
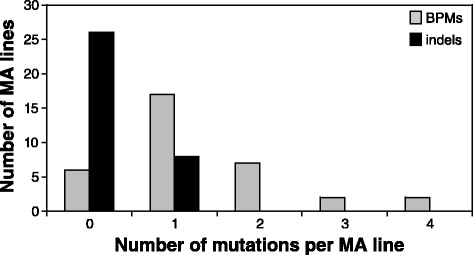
Distribution of number of BPMs and indels accumulated in wild-type MA lines

After 2500 generations, the genomes of the 36 *P. aeruginosa* MA lines were sequenced to an average coverage of 58.5X (Additional file 1). While we were initially interested in examining the effect of genetic background (Additional file 2) on the rate and spectrum of mutations, the mean number of mutations per MA line did not differ significantly between the four wild-type groups with different founder genotypes (F = 0.68, *p* = 0.57) so the data for these genotypes were pooled. Each of the 34 wild-type lines accumulated an average of 1.29 (SE = 0.18) base-pair mutations (BPMs; Fig. 1), which translated into a rate of 7.92 × 10^−11^ BPMs per nucleotide per generation (or 5.18 × 10^−4^ BPMs per genome per generation; Table 1). An average of 0.24 (SE = 0.07) indels accumulated per line, corresponding to an average rate of 1.44 × 10^−11^ indels per nucleotide per generation. In comparison, the mutator lines, which suffered from MMR deficiencies, accumulated an average of 0.14 BPMs and 0.022 indels per genome per generation. These values corresponded to “raw” mutations rates that were 267- and 230-fold greater than wild-type BPM and indel rates, respectively (Table 1).

**Table 1:** Accumulated mutations and mutation rates

MA lines	No. of lines	No. of BPM	BPM per line	BPM rate per genome	BPM rate per nucleotide	No. of indels	Indels per line	Indel rate per genome	Indel rate per nucleotide	No. of mutations	Mutations per line	Combined mutation rate per genome	Combined mutation rate per nucleotide
Wild-type	34	44	1.29	5.18 × 10^−04^	7.92 × 10^−11^	8	0.24	9.41 × 10^−05^	1.44 × 10^−11^	52	1.53	6.12 × 10^−04^	9.36 × 10^−11^
Mutator	2	690	345.00	1.38 × 10^−01^	2.11 × 10^−08^	108	54.00	2.16 × 10^−02^	3.30 × 10^−09^	798	399.00	1.60 × 10^−01^	2.44 × 10^−08^

### Neutral mutation accumulation

In the absence of selection, mutations should occur at random across the genome and so not be biased by, for example, differences between selection on coding versus non-coding regions or non-synonymous versus synonymous codon positions. We evaluated the effectiveness of our experimental procedure at reducing the influence of selection by examining the distribution of mutations across these two features of genomic architecture. Pooling the wild-type and MMR-deficient lines, 88.7 % of the observed BPMs (651 of 734) occurred within coding regions, which is not significantly different than what is expected by chance given that ~89.6 % of the PA14 genome is within protein-coding sequence (Chi-square test, χ^2^ = 0.35, *p* > 0.56). Based on codon usage data for the 5892 genes in the PA14 genome, 74.7 % of positions within coding DNA are non-synonymous positions. In the MA lines, 72.4 % of the observed BPMs in coding DNA (471 of 651) were non-synonymous, which is not significantly different than what is expected by chance (Chi-square test, χ^2^ = 0.89, *p* > 0.35). Consistent with other MA-WGS studies [4, 19, 20], our results suggest that mutations accumulated in a nearly neutral fashion, with little influence or bias from selection.

### Substitution spectrum and mutational bias

The observed rates for the different classes of substitutions were used to quantify the underlying mutational biases, namely, G:C versus A:T sites and the relative frequency of transitions versus transversions. The *P. aeruginosa* genome has a fairly high GC content (~67 %), so mutations are ~2X more likely to occur in a G:C position than an A:T position, by chance alone. We therefore normalized all class-specific substitution rates over the number of respective positions in the genome.

**Fig. 2:**
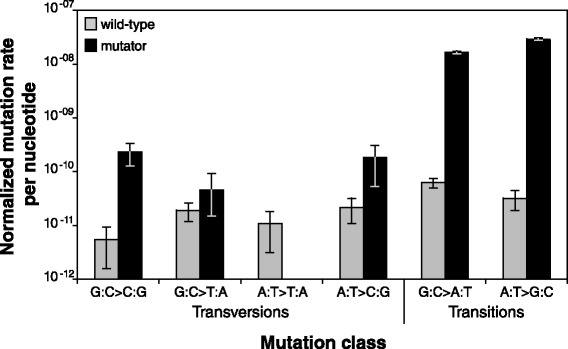
Normalized mutation rates for each mutation class. Error bars indicate standard error

The normalized mutation rate for G:C sites in wild-type lines was 8.69 × 10^−11^ per nucleotide per generation, which was 1.36X greater than that for A:T sites (6.41 × 10^−11^; Table 2). As expected, transitions made up the majority (65.9 %) of the substitutions and were 1.93X more common than transversions. Over three-quarters (79.3 %) of transitions were the G:C > A:T type, and the rate of G:C > A:T transition was 1.95X greater than that for A:T > G:C transition. For transversions, the highest rates were for the G:C > T:A and A:T > C:G types (Fig. 2, Table 2). Note that only 15 transversions were observed in wild-type lines, so these trends are based on a small, but presumably representative, sample.

**Table 2:** Proportions and rates for mutation classes

		Proportion		Normalized mutation rate per nucleotide (x10-11)		
	Mutation class	Wild-type	Mutator	Wild-type	Mutator	Fold increase in rate
All	G:C > N:N	0.727	0.528	8.69	1679.56	193.3
	A:T > N:N	0.273	0.472	6.41	2959.35	461.7
Tranversions	G:C > C:G	0.045	0.007	0.54	23.07	42.7
	G:C > T:A	0.159	0.001	1.90	4.61	2.4
	A:T > T:A	0.045	0.000	1.07	0.00	0.0
	A:T > C:G	0.091	0.003	2.14	18.16	8.5
Transitions	G:C > A:T	0.523	0.519	6.24	1651.88	264.7
	A:T > G:C	0.136	0.470	3.20	2941.19	919.1

The effect of a faulty MMR system, which was a 267X increase in overall substitution rates, disproportionately impacted A:T sites: these sites experienced a 462X rate increase whereas G:C sites increased by only 193X. The discrepancy was even more pronounced when comparing transitions and transversions (Fig. 2, Table 2), the rates for which increased an average of 592X versus 13X, respectively. The MMR system thus appears to be much more effective at correcting mismatches that lead to transitions than those that lead to transversions. Transitions comprised 98.8 % of all mutations in mutator lines, with the highest transition rate being for the A:T > G:C class, which was 919X higher than wild type lines.

### Insertion/deletions (indels)

We observed just 8 indels in the 34 wild-type MA lines, corresponding to a mutation rate that was one-fifth of that of BPMs (Table 1). The majority of indels (7/8) were associated with regions of repetitive DNA with repeat motifs ranging in length from 1 to 9 bases. In all cases, the number of repeats changed by only a single unit, with +1 unit insertions being more common then −1 unit deletions.

Deficiencies in MMR increased the overall indel rate by 230X, with insertions being 3.32X more common than deletions. Mean indel size was only 1.11 (SE = 0.04) bases and was substantially smaller than that for the wild-type lines (4.88, SE = 1.16). All 108 indels in the MMR-deficient lines were within stretches of repeated single nucleotides, with 96.3 % (104) in homopolymeric runs of G:C base pairs despite only 75.2 % being expected from the composition of the genome (Chi-square test, χ^2^ = 19.92, *p* < 0.0001). This significant over-abundance of G:C-based indels indicates that G:C homopolymers are more mutable than A:T homopolymers during DNA replication. Only one indel in the wild-type lines was in a homopolymer, so we cannot determine if this strong bias for G:C indels is altered by a functional *P. aeruginosa* MMR system.

**Fig. 3:**
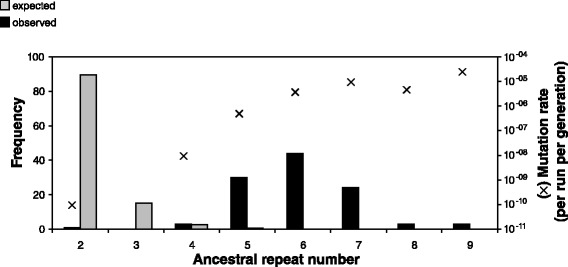
Indel frequencies and mutation rates in homopolymers in MMR-deficient MA lines

Indels in MMR-deficient lines were most common in homopolymers that were 6 bp in length (mean length 5.99 bp, SE = 0.10). This size class is strongly over-represented, considering that only 0.09 % of homopolymers in the genome are 6 bp long but 40.7 % of all indels were in this class (Fig. 3). More generally, homopolymers of 5 bp or longer were highly enriched for indel mutations, compared to that expected by chance. We calculated the per run indel rate for the different ancestral repeat numbers and found a positive relationship (Fig. 3), indicating that longer homopolymers have higher mutation rates.

### Leading/lagging strand bias

**Fig. 4:**
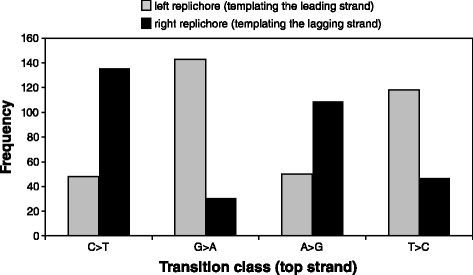
Frequencies for transition classes in the left and right replichore (called in reference to the top strand). Data are from the MMR-deficient MA lines. Observed values are significantly different from expected, for all classes (*p* < 0.0001)

Replication of double-stranded DNA is asymmetric because DNA synthesis must proceed in a 5’-3’ direction, resulting in continuous synthesis of the so-called ‘leading’ strand and discontinuous synthesis of its complementary ‘lagging’ strand. A consequence of bidirectional replication of circular genomes is that the two replichores differ in terms of which DNA strand (top or bottom) acts as the template for leading or lagging strand synthesis. If leading and lagging strand synthesis have different mutational biases, then these biases would occur reciprocally in each replichore. We observed these reciprocal patterns in transition data from our MMR-deficient lines, the only data set large enough to assess such biases. In the right replichore, G:C > A:T transitions were 4.39X more common when C rather than G was in the conventionally reported 5'-to-3' strand (top strand), and A:T > G:C transitions were 2.32X more common when A rather than T was in the top strand. In the left replichore, the reciprocal patterns were observed, with values of 0.34X and 0.42X, respectively (Fig. 4). These differences were significant for all transition classes (Chi-square, *p* < 0.0001), indicating there was a leading/lagging strand mutational bias.

To examine the leading/lagging strand bias more closely, we pooled the counts from each replichore and combined functionally equivalent site classes (Table 3; e.g., G templating the lagging strand in the right replichore is equivalent to C templating the leading strand in the left replichore). Overall, G:C > A:T transitions were 3.53X more common when G templated the leading strand rather than the lagging strand. Conversely, A:T > G:C transitions were less common (0.43X) when A templated the leading strand rather than the lagging strand. This strong leading/lagging strand mutational bias was significant for both types of transitions (*p* < 0.0001, Table 3). A leading/lagging strand bias was also evident for indels in the MMR-deficient lines. Indels in homopolymers of G:C were 4.20X more common when G templated the lagging strand rather than the leading strand.

**Table 3:** Patterns of leading/lagging strand mutational bias

		G:C > A:T		A:T > G:C	
		G templating the leading strand^a^	G templating the lagging strand^a^	A templating the leading strand^a^	A templating the lagging strand^a^
MMR-deficient MA lines	Expected	173	185	166	158
	Observed	**279**	79	97	**227**
	Observed Leading/Lagging	3.53		0.43	
	Chi-square value	67.42		30.47	
	p value	<0.0001		<0.0001	
Wild-type MA lines	Expected	12	13	4	4
	Observed	**15**	10	**6**	2
	Observed Leading/Lagging	1.50		3.00	
	Chi-square value	0.73		1.07	
	p value	>0.39		>0.30	
Comparative genomics of clinical isolates	Expected	16364	16384	8509	8511
	Observed	**17794**	14954	**8837**	8183
	Observed Leading/Lagging	1.19		1.08	
	Chi-square value	125.12		12.65	
	p value	<0.0001		<0.0004	

Observed values that are greater then expected are shown in bold. Data from both replichores were combined

^a^Functionally equivalent transition types were combined. For example, G templating the leading strand in the right replichore is functionally equivalent to C templating the lagging strand in the left replichore

To what extent are these biases corrected by a functional MMR system? If mismatches are repaired with similar efficiencies on both leading and lagging strands, then these pre-mutation biases would carry over as post-MMR substitution biases. The wild-type, MMR-proficient MA lines retained the G:C > A:T pre-mutation transition bias however the A:T > G:C pre-mutation transition bias was reversed (Table 3). Note that the MMR system is very efficient at fixing transitions, so our wild-type sample size was low and none of these differences were statistically significant. To increase our sample size, we leveraged transition data from a genome alignment of clinical *P. aeruginosa* isolates reported in our previous comparative genomics study [21]. Analyses of 49,768 transitions from natural population data (Table 3) showed that, much like the wild-type MA lines, the G:C > A:T pre-mutation transition bias was still present and the A:T > G:C pre-mutation transition bias was reversed. Note, however, that the intensity of the leading/lagging bias in the population data is less than that found in the MA lines. These data from natural isolates are assumed to represent wild-type MMR function, although it is likely that at least some isolates have experienced a history of MMR deficiency. While the effects of natural selection in these populations cannot be discounted, there is no obvious reason to expect a bias towards a particular transition type. Our results therefore suggest that a functional MMR system in *P. aeruginosa* counteracts the leading/lagging strand pre-mutation bias, but does so more efficiently for the A:T > G:C transition class.

### Mutation distribution along genome

**Fig. 5:**
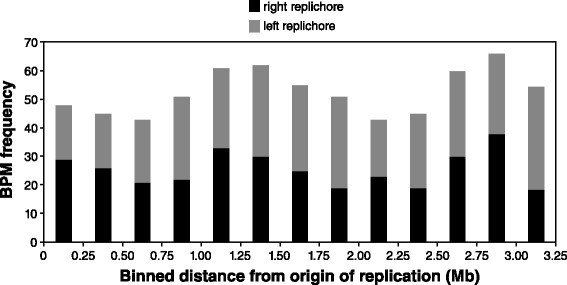
Frequency of BPMs as a function of distance from origin of replication. Data are from MMR-deficient MA lines pooled

Chromosomal position could create a mutation rate bias that results in a non-random distribution of mutations along the length of the circular genome. We found that the cumulative distribution of mutations in wild-type lines deviated from a linear relationship with genome position (Additional file 3), with the largest cluster of mutations appearing between the 3 MB and 4 MB marks near the terminus of replication (position 3,175,707). This result suggested a relationship between mutation rate and location in the replichore. Upon closer examination, there appeared to be a somewhat bi-modal distribution for both the wild-type (Additional file 4) and MMR-deficient (Fig. 5 and Additional file 4) data sets when the number of mutations was plotted against distance from origin of replication. Peaks in the distribution were near the terminus (2.75–3.0 MB) and intermediate between the origin and terminus (1.25–1.50 MB), and were evident in both replichores (Fig. 5). Finding this wave-like distribution in both wild-type and MMR-deficient lines suggests it is caused by biases in underlying raw mutation rates, rather than biases in rates of mismatch repair.

**Fig. 6:**
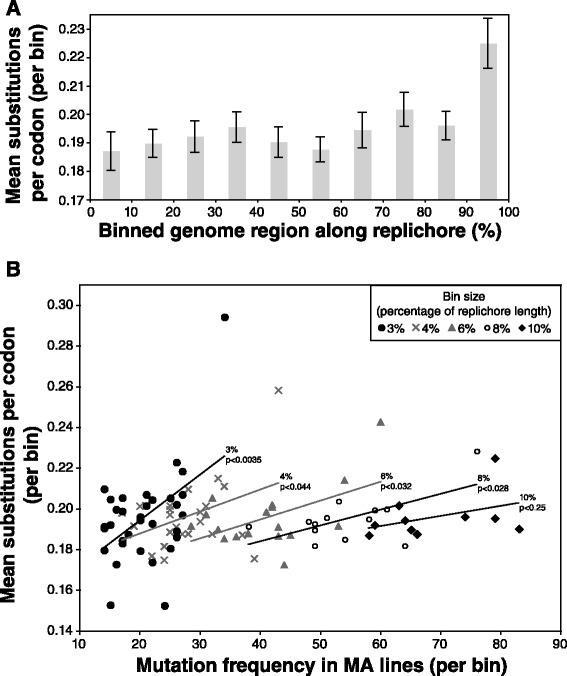
Diversity in core genes in natural populations of *P. aeruginosa*. Diversity data are for 2827 core genes from clinical isolates analyzed in ref. 21. **a** Mean substitutions per codon for core genes, binned by position along replichore (10 % increments, both replichores pooled). Error bars indicate standard error. **b** Relationship between diversity in core genes in natural isolates and the number mutations observed in MMR-deficient MA lines. Replichores were divided into bins of set proportional sizes and both measures were calculated for each bin. Analyses were performed with different percentages of replichore length in each bin (3 % to 10 %). Significance of positive correlation is indicated on trendlines

We examined existing data on genomic variation in clinical *P. aeruginosa* isolates for evidence of the residual effects of a mutation distribution bias in natural populations. For each of the 2827 core genes from a 32-genome alignment of *P. aeruginosa* isolates (see ref. [21]), we determined its location in the replichore and the number of substitutions per codon. When the replichores were binned into 10 % increments and the diversity data were averaged (Fig. 6a), we observed a wave-like pattern that was similar to that observed for the MA lines (Fig. 5 and Additional file 4). A pattern of high diversity near the terminus of replication was very pronounced, particularly within the last 10–30 % of core genes. We repeated this analysis with a range of bin proportions (3, 4, 6, 8, and 10 %) and found significant positive correlations between natural population diversity and MA-line mutation frequency (Fig. 6b). These data suggest that the mutation distribution bias during DNA replication may affect the levels of diversity observed along the chromosome in natural bacterial populations.

### Context-dependent mutation rates

**Fig. 7:**
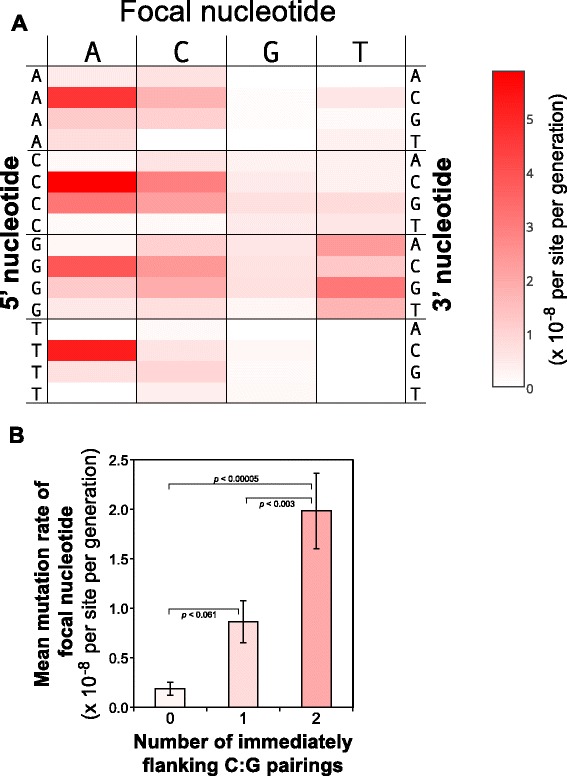
Context-dependent mutation rates in MMR-deficient MA lines. **a** Heat map of context-dependent mutation rates of focal nucleotides (lagging stand orientation). Focal nucleotides are along the top, whereas 5’-flanking and 3’-flanking nucleotides are on the left and right, respectively. **b** Effect of the number of immediately flanking (5’ or 3’) C:G pairings on the mutation rate of focal nucleotides. Error bars indicate standard error

We have shown that large scale differences such as leading and lagging strand synthesis and chromosomal position can influence mutation rate, however, small scale differences in the local nucleotide environment may also have an effect. Based on the mutator MA line data, we calculated the context-dependent mutation rates for the center (focal) nucleotide of each of the 64 possible nucleotide triplets. Rates were similar between replichores so we present the rates from both replichores combined (top, lagging strand orientation, Fig. 7, Additional file 5). Context-dependent mutation rates of focal nucleotides varied drastically and were affected by the characteristics of immediately adjacent nucleotides (5’- and 3’-flanking, both *p* < 0.0001, Additional file 6). Even for focal nucleotide A, which had the highest mean mutation rate (1.77 × 10^−8^), triplet-specific rates ranged from zero to 5.86 × 10^−8^ per generation. For example, when comparing the 16 triplets with a focal A (5’-N[A]N-3’), the mutation rate was greatest when the 3’-flanking nucleotide was C (5’-N[A]C-3’), regardless of which of the four possible 5’-flanking nucleotides were present (Fig. 7a). Overall, a C:G pairing in the 5' and/or 3'-flanking position was associated with higher mutation rates of the focal nucleotide (Fig. 7b). These results clearly demonstrate that local nucleotide context has a large influence on mutation rate.

## Discussion

### Genome-wide mutation rates

Over 20 years ago, a review of available data for short-term, locus-specific mutation rates concluded that DNA-based microbes have an approximately constant wild-type BPM rate of 3.3 × 10^−3^ per genome per generation [22]. Our calculated genome-wide mutation rate of 0.52 × 10^−3^ per genome per generation for *P. aeruginosa* is ~6x lower than Drake’s estimate, but is very comparable to those from other MA-WGS studies in bacteria and the unicellular eukaryote *S. cerevisiae* (Table 4). Considering the potential variation introduced by organism-specific and experimental differences, compounded by the stochastic nature of the mutational process itself, it is remarkable that these estimated mutation rates are even within an order of magnitude from each other.

**Table 4:** Comparison of genome-wide mutation rate estimates

	Wild type		MMR-deficient		
Organism	per genome per generation	per nucleotide per generation	per genome per generation	per nucleotide per generation	Reference
*P. aeruginosa*, mutation accumulation	0.52 × 10^−3^	0.79 × 10^−10^	0.14	2.11 × 10^−8^	this study
*P. aeruginosa*, mutation accumulation	n/a	n/a	0.18	2.95 × 10^−8^	ref. [20]
*E. coli*, mutation accumulation	0.92 × 10^−3^	1.99 × 10^−10^	0.13	2.75 × 10^−8^	ref. [4]
*E. coli*, synonymous sites	0.41 × 10^−3^	0.89 × 10^−10^	n/a	n/a	ref. [61]
*S. typhimurium*, mutation accumulation	3.4 × 10^−3^	7.0 × 10^−10^	0.09	2.0 × 10^−8^	ref. [19]
*S. cerevisiae*, mutation accumulation	5.84 × 10^−3^	4.8 × 10^−10^	0.90	7.4 × 10^−8^	ref. [62]
*S. cerevisiae*, mutation accumulation	4.01 × 10^−3^	3.3 × 10^−10^	n/a	n/a	ref. [3]

A functional MMR system plays an important role in correcting DNA replication errors. Nucleotide changes that occur during DNA replication result in mismatches between the template and daughter strand. Most of these mismatches (pre-mutations) are identified and returned to the ancestral state by the MMR system of the cell, but a small proportion are resolved to the derived state and become true mutations. In *P. aeruginosa*, deficiencies in MMR increased the BPM rate by 267-fold, a value that corresponds to a mutation rate similar to that reported in other MMR-deficient strains in other bacterial species (0.09 to 0.18 mutations per genome per generation, Table 4). Rate comparisons for *P. aeruginosa* indicate that 99.6 % of raw pre-mutations (BPMs and indels) are corrected by the MMR system before they become stably incorporated into the genome. The net mutation rate therefore represents a balance between the error-prone DNA replication process and the highly efficient error-repair mechanisms.

Although the genome-wide indel rate increased by 230-fold in MMR-deficient *P. aeruginosa* lines, these increases were non-randomly localized to very specific sub-sequences of the genome, mainly homopolymers between 5 and 7 base-pairs in length (Fig. 3). Given that homopolymers represent only a small proportion of the genome, the per run rate of mutation could be very high. For example, a 9 bp homopolymer experienced a mutation, on average, every 8 generations (0.13 per generation per run). We found that longer homopolymers had higher mutation rates (Fig. 3), likely due to the susceptibility of long homopolymer runs to slipped-strand mispairing, the primary mechanism of mutation in regions of simple sequence repeats [23]. Longer stretches of repeated sequence pose more of a problem to polymerase than do shorter stretches, so longer alleles are more prone to the pairing of misaligned repeats, which leads to the insertion/deletion of repeat units. Notably, indel rates in *P. aeruginosa* reached a plateau at 7 bases and above, matching the relationship between homopolymer length and mutation rate observed in natural hypermutator isolates of *P. aeruginosa* [24].

### The origins of GC-biased genome content

The equilibrium GC content of *P. aeruginosa* genomes is ~67 %, the highest for any bacteria to which the MA-WGS approach has been applied. Nevertheless, our results indicate that, as in other systems, the mutational process is universally biased towards AT [25, 26]. In highly GC-rich species, this universal bias towards AT must be overcome by an opposing bias towards GC. In wild-type *P. aeruginosa* MA lines, the normalized rate of mutation from GC to AT base-pairs was 1.52X greater than that for AT to GC base-pairs. In MMR-deficient lines, the greatly increased rate of A:T > G:C transitions (Table 2, Fig. 2) led to an overall bias towards GC (1.79X), the opposite of that observed in wild-type lines. The *net* mutational bias towards AT in wild-type lines indicates that a functional MMR system efficiently counter-acts the *raw* mutational bias towards GC, which, if left unchecked, would lead to incredibly rapid increases in GC content of the genome. Surveys of a range of bacterial genomes have found little correlation between the presence of specific mismatch repair genes and the equilibrium GC content of the genome [27]. We note, however, that within a bacterial species, different strains have different levels of MMR functionality [10, 28].

We hypothesize that the equilibrium GC content of a recombining bacterial species may be a function of the prevalence of MMR-proficient strains, which tend to decrease GC content, and MMR-deficient mutator strains, which tend to increase GC content. If this is the case, then species with higher proportions of MMR-deficient mutators would have higher equilibrium GC content. Consistent with this hypothesis, the raw mutation and MMR biases observed in *P. aeruginosa* and *E. coli* are very similar, but the GC content of *P. aeruginosa* is higher (67 % versus 51 %) and mutator strains appear to be more common in *P. aeruginosa* than *E. coli*. Although comprehensive species-wide surveys are lacking, studies focusing mainly on pathogenic isolates indicate mutator prevalence is up to 54 % in *P. aeruginosa* [10] but only 1–12 % in *E. coli* [29, 30]. It is worth pointing out, however, that our data set does not allow us to exclude other potential explanations such as selection for increased synonymous GC content, biased gene conversion, or translational selection [31–33].

### Substitution spectrum and context dependency

The substitution spectra for wild-type and MMR-deficient lines (Fig. 2) differed substantially, demonstrating that the MMR system corrects different classes of pre-mutations with different efficiencies. For example, MMR deficiency allowed G:C > T:A transversion rates to double, whereas A:T > G:C transition rates increased by nearly three orders of magnitude (919X). The class-specific mutational biases observed in *P. aeruginosa* are nearly identical to those reported for *E. coli* [4, 20]. In both of these species, for example, G:C > A:T transitions were the most common BPMs in wild-type lines and A:T > G:C transitions were the most common BPMs in MMR-deficient lines. In contrast, the results from the MA-WGS study with *Salmonella typhimurium* [19] were very different. The discrepancies in mutational biases among bacterial species may be explained by differences among experiments in the genetic cause of MMR deficiency. The mutator phenotype in *S. typhimurium* was achieved by deletion of five genes (*ung*, *vsr*, *mug*, *mutM*, *mutY*) involved in major DNA repair systems that correct common spontaneous mutations caused by oxidized and deaminated bases [34]. DNA synthesis past oxidized guanines (8-hydroxyguanine), an abundant form of oxidatively damaged DNA, typically leads to G:C > T:A transversions [35]. Consistent with this mechanism, 91 % of the BPMs in these mutator *S. typhimurium* lines were G:C > T:A transversions. In contrast to *S. typhimurium*, the *P. aeruginosa* and *E. coli* mutators both were caused by knockouts in the methyl-directed mismatch repair system (*mutS* and *mutL* genes, respectively; [4, 20, 36]). The mutS and mutL genes function together in the same MMR pathway, so it is not surprising that the mutational bias in these knockouts were very similar, despite being in different bacterial species.

We also found evidence for context-dependent mutation rates, with higher mutation rates for focal nucleotides that were flanked on either or both sides by a C:G base pair. The mechanism responsible for this effect likely involves the impact of interactions between adjacent bases, known as base-stacking, on the stability of double-stranded DNA. A:T pairings are more destabilizing than C:G pairings [37] and lead to greater deformation of the double helix when a mismatch is present. The reduced helix deformation by C:G-flanked mismatches may reduce their probability of mismatch detection by the proofreading activity of the DNA polymerase, allowing these mismatches to evade correction and be retained. The positive correlation between the observed mutation rate and the number of immediately flanking C:G pairings seen in Fig. 7b is consistent with this interpretation, suggesting that base-stacking may explain much of the variation in context-dependent mutation rates observed here and in other studies [4].

### Leading/lagging strand bias

We found clear evidence for a strong, leading/lagging mutational bias in our MMR-deficient MA lines and natural *P. aeruginosa* population data. For our MMR-deficient MA lines, C and A were most mutable when templating the lagging strand, whereas G and T were most mutable when templating the leading strand (Table 3, Fig. 4). In contrast, our *P. aeruginosa* population data showed that C and T were most mutable when templating the lagging strand, whereas G and A were most mutable when templating the leading strand. The cause of leading/lagging bias is unclear but could stem from differences in the replication enzymes associated with leading (continuous) versus lagging (discontinuous) synthesis, or from the duration of time one or the other strand is in a single-stranded state. Our data cannot distinguish directly between these mechanisms, although some indirect evidence suggests that time spent in the single-stranded state is not a major contributor to this pattern. The leading strand is in a single-strand state for a longer period of time than the lagging strand, making it more susceptible to deamination-based mutation [38] and lowering the overall fidelity of replication [39]. Deamination disproportionately impacts cytosine bases [40], leading to the formation of uracil, which pairs with adenine during DNA replication to create a C > T transition. Thus, C would be most susceptible to transition when templating the leading strand. The patterns of differential mutability in leading/lagging strands observed here for *P. aeruginosa*, which match closely those observed in *E. coli* [4], are not consistent with this prediction. It is further worth noting that indels showed a leading/lagging strand bias as well, though in the opposite direction than for BPMs. Mutations in G:C homopolymers were 4.20X more common when G templated the lagging vs. leading strand. This finding demonstrates that differences between DNA replication in the leading and lagging strand have mechanism-specific effects on the resulting mutational spectrum.

### Heterogeneity in mutation rates across the genome

DNA replication in *P. aeruginosa* occurs bidirectionally, being initiated at a single origin and proceeding in opposite directions around each half of the chromosome until terminating when the replication machineries meet at the approximate midpoint of the chromosome. When the replication machinery of opposing replication forks collide, the disruption of DNA synthesis may allow errors to occur, thereby increasing the mutation rate near the terminus of replication [41]. Previous studies have found either that mutation rates tend to increase approximately linearly with distance from the origin of replication [42–44] or peak at intermediate positions between the replication origin and terminus (*Salmonella enterica* [45], *E. coli* [5]). By contrast, we have found a bi-modal distribution of mutations with terminal and intermediate peaks in *P. aeruginosa*, a result consistent with that observed in MMR-defective *E. coli* [5]). The underlying cause of this relationship is unknown, but observation of this pattern in two different bacterial species suggests a shared mechanism of replication bias that warrants further study.

The observation of parallel evolution, the repeated evolution of the same phenotype or genotype in independently evolving populations, is often attributed to strong selection. However, the probability of parallel evolution can be influenced by any factor that affects the amount of genetic variation available to selection [46], such as mutation rate. For example, some genomic regions may be more highly mutable than others and so are more likely to generate variants that are eventually selected during adaptive evolution. The relative contributions of selection and mutation to the observation of parallel evolution remain unresolved, but our data help shed some light on the role of mutation in generating standing variation. We found a positive correlation between region-specific mutation rates, as measured from our MA experiment, and observed levels of polymorphism amongst our collection of clinical isolates of *P. aeruginosa*. While the significance of these relationships is mainly driven by a small number of data points, and depends on how finely binned the genome is, the slope remains positive across all bin sizes. Given that regions of high mutation rate may be more likely to generate adaptive variation that can be selected in natural isolates, these results underscore the importance of accounting for heterogeneity in mutation rates across the genome when interpreting patterns of parallel evolution.

### Implications for pathoadaptation

Perhaps one of our most important findings is how different the mutational patterns are between wild-type and mutator strains. Hypermutable strains with deficiencies in some capacity of MMR are often found in natural populations of bacteria, with relatively high proportions in species that colonize the CF lung environment (*P. aeruginosa* [47], *Staphylococcus aureus* [48], *Haemophilus influenzae* [49]). MMR-deficient strains are more prevalent in samples of *P. aeruginosa* from chronic infections than in samples from environmental or acute sources [10, 28], suggesting that increased mutation rates may confer an adaptive benefit within the CF lung. Clinical and laboratory studies have found that mutator strains often show increased evidence of adaptation in several phenotypes relevant to this pathosystem, such as antibiotic resistance, biofilm formation, oxidative stress resistance, and competitive fitness [12–15, 50–53]. The apparent increased adaptability by mutators may be facilitated by an increased rate at which beneficial mutations arise [11], as well as a shift in the underlying spectrum of mutations that are available for selection [54]. If particular adaptive pathways are accessible only by specific types of mutations, mutators may be able to access them more readily because those types of mutations are relatively more common.

A prime example of a mutational spectrum shift in mutators is the 230-fold increase in indel rate that was localized to within homopolymeric tracts. We found that hypermutability caused the proportion of indels in G:C homopolymers to increase from 0 to 96.3 %. Given that single base-pair indels predominate, nearly every indel in a homopolymer within a protein-coding gene will result in a frameshift mutation and disruption of protein function. An important point about homopolymers is that their high rate of mutation via slipped-strand mispairing means back-mutations (reversions) that restore the original reading frame would also be common. Therefore, these homopolymers form the basis for a genetic switch that can rapidly alternate the gene between a functional and non-functional state, which could be beneficial under conditions with fluctuating selection pressures (e.g. acute vs chronic infection). In *P. aeruginosa*, for example, loss-of-function mutations in genes associated with antigenicity, motility, secretion activities, or biofilm formation may be pathoadaptive by helping the bacteria to evade host immune detection or response [9, 21, 24, 55, 56]. Thus, hypermutation itself may be adaptive by allowing gene-localized homopolymers to be a mutational mechanism for regulating the contingent expression of virulence factors [57].

## Conclusions

Mutation is the ultimate source of all genetic variation but our understanding of the factors that govern the rate and spectrum of mutations produced, and how they contribute to standing variation in natural populations, remains limited. To help address this issue, we used whole-genome sequencing to reveal the genome-wide rate, spectrum, distribution, leading/lagging bias, and context-dependency of spontaneous mutations that arose over 90,000 cumulative generations of growth in both wild type and MMR-deficient mutator lines of the opportunistic human pathogen *P. aeruginosa*. Our leading results are: (1) wild-type BPM rates are ~5 x 10^−4^ per genome per generation, approximately 6X lower than previously thought; (2) deficiencies in MMR increases the mutation rate by over two orders of magnitude; (3) there is a mutational bias towards decreased GC content in wild-type strains which appears to be counteracted by a reverse bias in MMR-deficient strains; (4) there is substantial heterogeneity in mutation rates across the genome contributed by various sources including leading/lagging strand bias, distance from the origin of replication, length of homopolymer runs, and local nucleotide context; and (5) there is a positive correlation between mutation rates along the genome and extant levels of polymorphism among clinical isolates. Taken together, these results collectively provide a more complete view of how several mechanisms of mutation, mutation repair, and bias act simultaneously to produce the net mutational patterns.

## Methods

### Strain information and propagation

Founder strains of *P. aeruginosa* were PA14, or were derived from PA14 as described in ref. 58. The genome sequences of the founder strains smB3, smB4, and smC3 differ from PA14 at one to three mutations (Additional file 2), and all of these strains are wild type in regards to mutation rate. Founder strain smA5 differs from PA14 at 30 mutations, one of which is a 11-bp deletion causing a frameshift in the mismatch-repair gene *mutS*, conferring a mutator phenotype.

MA lines were initiated by creating ten replicates of each of the five founder strains. Lines were assigned numerical suffixes reflective of the founder strain, for example, PA14 MA lines were named PA14-1 to PA14-10. Populations were propagated on Lysogeny Broth agar plates (peptone 10.0 g/L, yeast extract 5.0 g/L, NaCl 5.0 g/L, 1.5 % agar) by harvesting cells from the periphery of a single colony and streaking them onto a new plate to start the next growth cycle for a total of 90 cycles. To avoid bias, we sampled the distinct colony closest to the end of the streak trace, regardless of colony size or morphology. The standard growth cycle was 24 h of growth at 37 C, with occasional (14 of 90) growth cycles at a lower temperature of 22 C. Fourteen of the fifty MA lines were lost due to transfer errors, contamination, archive failure, or lineage ambiguities. The majority of lost lines (8 of 14) were mutator lines that became contaminated. Regardless, all analyses presented here are restricted to the 36 MA lines whose fidelity could be confirmed by colony phenotype data and genome sequence.

### Number of generations

For each endpoint MA line, the total number of viable cells per colony was determined by harvesting an entire colony, re-suspending in buffer, and dilution plating on solid Lysogeny Broth medium. Colonies were initiated from single cells, so the number of generations per growth cycle was estimated as log2 of total cell number. The mean number of generations per MA line was 2460 (SE = 53), although this likely represents an underestimate as a small fraction of cells will be inviable. To account for this fact, we have conservatively used 2500 as the number of generations for each line.

### Genome sequence analyses

Single colonies from the endpoints of MA lines were isolated, grown in Lysogeny Broth, and genomic DNA was extracted using a Qiagen DNeasy Blood & Tissue Kit. Sequence data were obtained from 100-bp, paired-end reads using the Illumina Hi-Seq platform (BC Cancer Agency, Genome Sciences Centre, Vancouver, Canada). Sequence data obtained for the MA lines was archived in NCBI Short Read Archive under BioProject PRJNA302277. Raw sequence data for the founder genotypes were from [58]. A modified version of the bioinformatics pipeline described in [59] was used for subsequent data analyses. In brief, reads were trimmed using Popoolation (ver. 1.1) with a phred quality threshold of 20 and a minimum retention length of 75 bp. Trimmed reads were mapped to the PA14 reference genome (NC_008463.1) using Novoalign (ver. 2.07). Single nucleotide polymorphisms and insertion/deletions (indels) relative to the reference were called using Samtools (ver. 0.1.19; minimum coverage = 3X), VarScan (ver. 2.3.5; minimum coverage = 3X, *p* > 0.95), and Pindel (ver. 0.2.4). Results from the different methods were compared for cross-verification of called variants. Genomic variation was annotated using SnpEff (ver. 3.3; minimum coverage = 5X, minimum base quality = 20). MA line mutations were identified by comparing the nucleotide states in the genomes of PA14, founder isolates, and the respective MA lines. For quality control, we verified that mutations specific to founder isolates were also called correctly in all descendant MA lines.

### Mutation rates

Mutation rate calculations assumed each MA line underwent 2500 generations. When necessary, mutation rates were normalized over the number of nucleotides analyzed, to give class-specific rates. Pooled standard errors were calculated by the average standard error of mutation rate across lines divided by the square root of the number of lines analyzed [60]. Similarly, context-dependent mutation rates of focal nucleotides in a triplet accounted for the number of those specific triplets in the genome. It cannot be determined which strand the mutations arose on, so context-dependent mutation rates are reported for triplets in the orientation found on the top strand of the right replichore (templating lagging strand synthesis).

### Polymorphism data from natural isolates

Data for naturally occurring transitions were extracted from a 32-genome alignment reported in our previous comparative genomics study of *P. aeruginosa* [21]. The direction of nucleotide change for transitions was determined by setting the ancestral state to that found in the basal clade of the phylogeny (PA14, PAb1, and PA3906). Data for core gene polymorphism was parsed from results of previous likelihood-based analyses of selection (PAML) on core genes (i.e., genes present in all 32 genomes). To account for differences in gene length, the metric of polymorphism used was the average number of substitutions per codon. Positional order of the core genes in the replichore was determined using the PA14 genome as a reference.

### Availability of supporting data

The short-read sequence data sets supporting the results of this article are available from the NCBI Short Read Archive (http://trace.ncbi.nlm.nih.gov/Traces/sra/) under BioProject PRJNA302277 and BioSample accessions SAMN04271187 – SAMN0427222.

## Additional files

Additional file 1: Table S1. Genome sequencing summary statistics. (DOC 36 kb) Additional file 2: Table S2. Founder genotypes and their mutations relative to the PA14 genome. (DOC 109 kb) Additional file 3: Figure S1. Cumulative distribution of BPMs in the genomes of wild-type MA lines. (PDF 276 kb) Additional file 4: Figure S2. Frequency of mutations as a function of distance from origin of replication. a) BPMs from wild-type MA lines pooled with both replichores combined. b) Indels from MMR-deficient MA lines pooled with both replichores combined. (PDF 302 kb) Additional file 5: Table S3. Context-dependent mutation rates of focal (center) nucleotides of 64 triplets (top, lagging strand orientation). Rates are per site per generation. (DOC 88 kb) Additional file 6: Table S4. Results of model for effect of variables on context-dependent mutation rates of focal nucleotide. (DOC 30 kb)

## Abbreviations

BPM: Base-pair mutation
CF: Cystic fibrosis
MA: Mutation accumulation
MMR: Mismatch repair
WGS: Whole-genome sequencing
