# Supplementary material for: The properties of spontaneous mutations in the opportunistic pathogen Pseudomonas aeruginosa
Source: BMC Genomics. 2016 Jan 5;17:27. doi: 10.1186/s12864-015-2244-3 (PMC4702332; doi:10.1186/s12864-015-2244-3)
Supplement: Additional file 1: Table S1. — Genome sequencing summary statistics. (DOC 36 kb) [file 12864_2015_2244_MOESM1_ESM.doc]

**Table S1 Founder genotypes and their mutations relative to the PA14 genome.**

| **Founder genotype** | **Number of mutations** | **Genome position, mutation** | **Effect on gene** |
| --- | --- | --- | --- |
| smB3 | 1 | 5432300, T -> A | *morA*, motility regulator (PA14_60870), codon 1155, L -> Q |
| smB4 | 2 | 5428225, 11 bp deletion | *nfxB*, transcriptional regulator (PA14_60860), codon 65, frameshift |
|  |  | 5430523, A -> C | *morA*, motility regulator (PA14_60870), codon 563, T -> P |
| smC3 | 1 | 5024315, C -> T | putative diguanylate cyclase (PA14_56280), codon 204, M -> I |
| smA5 | 30 | 1503203, 11 bp deletion | *mutS*, DNA mismatch repair protein (PA14_17500), codon 94, frameshift |
|  |  | 29 other mutations, see Wong et al. (2012) Supplemental Material for details |  |
