# Supplementary material for: The properties of spontaneous mutations in the opportunistic pathogen Pseudomonas aeruginosa
Source: BMC Genomics. 2016 Jan 5;17:27. doi: 10.1186/s12864-015-2244-3 (PMC4702332; doi:10.1186/s12864-015-2244-3)
Supplement: Additional file 2: Table S2. — Founder genotypes and their mutations relative to the PA14 genome. (DOC 109 kb) [file 12864_2015_2244_MOESM2_ESM.doc]

**Table S2 Genome sequencing summary statistics.**

| **Genome** | **Number of reads** | **Percentage of reads aligned** | **Mean fold coverage** | **Number of BPMs called** | **Number of indels called** |
| --- | --- | --- | --- | --- | --- |
| PA14-1 | 3039492 | 99.08 | 45.84 | 1 | 0 |
| PA14-2 | 4076586 | 99.20 | 61.54 | 1 | 1 |
| PA14-3 | 4233144 | 99.19 | 63.89 | 1 | 0 |
| PA14-4 | 2582882 | 99.04 | 38.92 | 1 | 0 |
| PA14-5 | 4556892 | 99.23 | 68.81 | 1 | 1 |
| PA14-6 | 4054466 | 98.92 | 61.05 | 1 | 0 |
| PA14-7 | 5087810 | 99.05 | 76.68 | 1 | 0 |
| PA14-8 | 2660294 | 99.16 | 40.14 | 0 | 1 |
| PA14-9 | 1624646 | 99.09 | 24.50 | 1 | 0 |
| PA14-10 | 4407962 | 99.22 | 66.56 | 1 | 0 |
|  |  |  |  |  |  |
| smB3-1 | 3276820 | 99.19 | 49.48 | 2 | 0 |
| smB3-2 | 4045126 | 99.20 | 61.07 | 1 | 0 |
| smB3-3 | 4445372 | 99.22 | 67.12 | 3 | 0 |
| smB3-4 | 4019808 | 99.16 | 60.67 | 4 | 0 |
| smB3-5 | 3440344 | 99.21 | 51.94 | 2 | 0 |
| smB3-8 | 4934076 | 99.11 | 74.41 | 1 | 0 |
| smB3-9 | 4072548 | 99.03 | 61.39 | 0 | 0 |
|  |  |  |  |  |  |
| smB4-2 | 4335892 | 99.23 | 65.46 | 1 | 0 |
| smB4-3 | 2827280 | 99.20 | 42.69 | 0 | 0 |
| smB4-4 | 4528468 | 99.16 | 68.35 | 0 | 0 |
| smB4-5 | 4549804 | 99.18 | 68.68 | 1 | 1 |
| smB4-6 | 4960270 | 99.20 | 79.76 | 4 | 0 |
| smB4-7 | 4167396 | 99.21 | 62.92 | 0 | 0 |
| smB4-8 | 4007492 | 99.21 | 60.51 | 2 | 0 |
| smB4-9 | 3206388 | 99.14 | 48.43 | 1 | 1 |
|  |  |  |  |  |  |
| smC3-1 | 4613902 | 98.96 | 69.49 | 2 | 1 |
| smC3-2 | 4427390 | 99.16 | 66.96 | 2 | 0 |
| smC3-3 | 5031478 | 99.16 | 75.91 | 1 | 1 |
| smC3-4 | 5344528 | 99.17 | 80.65 | 0 | 0 |
| smC3-5 | 2941548 | 99.25 | 44.45 | 2 | 0 |
| smC3-6 | 4230056 | 99.19 | 63.86 | 1 | 0 |
| smC3-7 | 4132184 | 99.19 | 62.36 | 3 | 0 |
| smC3-8 | 4725264 | 99.24 | 71.38 | 2 | 1 |
| smC3-10 | 4117874 | 99.24 | 62.19 | 0 | 0 |
|  |  |  |  |  |  |
| smA5-1 | 988210 | 98.98 | 14.90 | 356 | 64 |
| smA5-4 | 1448442 | 99.05 | 21.83 | 334 | 44 |
|  |  |  |  |  |  |
| MA line mean | 3865059.28 | 99.15 | 58.47 |  |  |
| Standard error | 173074.64 | 0.01 | 2.64 |  |  |
|  |  |  |  |  |  |
| PA14 founder* | 1296358 | 98.10 | 15.06 |  |  |
| smB3 founder* | 1536492 | 98.63 | 17.56 |  |  |
| smB4 founder* | 2945830 | 98.41 | 33.47 |  |  |
| smC3 founder* | 3061972 | 98.61 | 34.84 |  |  |
| smA5 founder* | 1819378 | 98.55 | 20.75 |  |  |
|  |  |  |  |  |  |
| Founder mean | 2132006 | 98.46 | 24.33 |  |  |
| Standard error | 298765.63 | 0.08 | 3.36 |  |  |

*sequence data for founder genotypes were 75-bp paired-end reads (data from Wong et al. 2012)
