# Supplementary figures and images for: The properties of spontaneous mutations in the opportunistic pathogen Pseudomonas aeruginosa
Source: BMC Genomics. 2016 Jan 5;17:27. doi: 10.1186/s12864-015-2244-3 (PMC4702332; doi:10.1186/s12864-015-2244-3)

**Figure S1: Cumulative distribution of BPMs in the genomes of wild-type MA lines.**

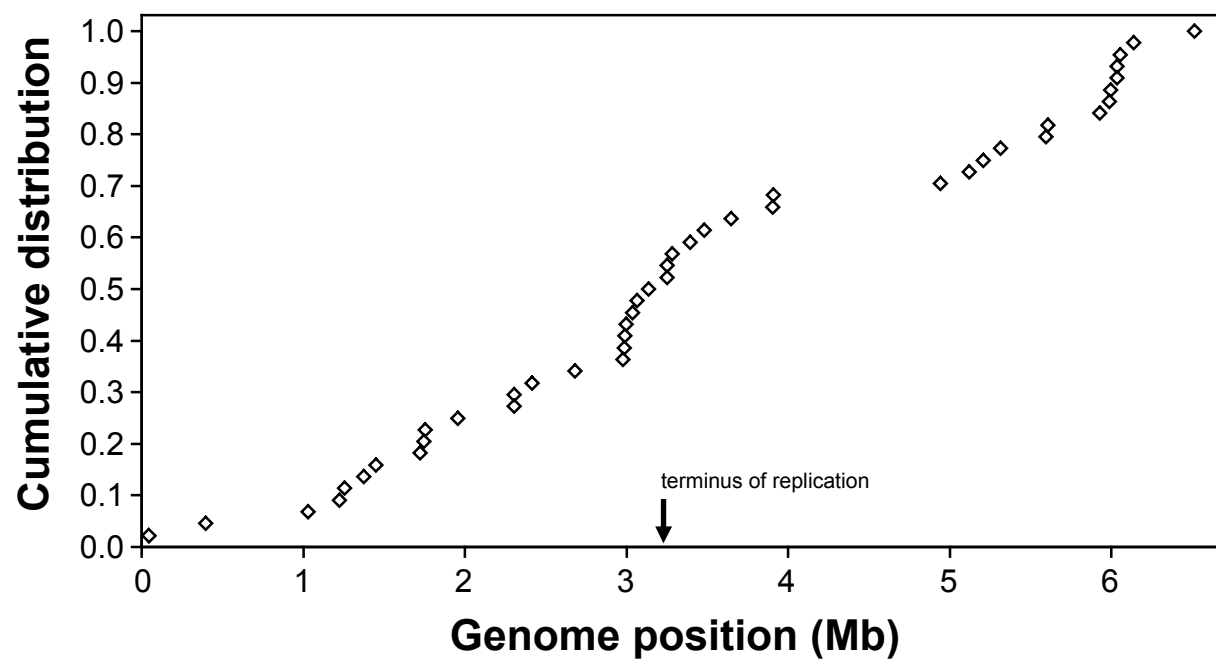

Supplement: Additional file 3: Figure S1. — Cumulative distribution of BPMs in the genomes of wild-type MA lines. (PDF 276 kb) [file 12864_2015_2244_MOESM3_ESM.pdf]
