# Supplementary material for: The properties of spontaneous mutations in the opportunistic pathogen Pseudomonas aeruginosa
Source: BMC Genomics. 2016 Jan 5;17:27. doi: 10.1186/s12864-015-2244-3 (PMC4702332; doi:10.1186/s12864-015-2244-3)

Figure S2: Frequency of mutations as a function of distance from origin of replication.
A) BPMs from wild-type MA lines pooled with both replichores combined.
B) Indels from MMR-deficient MA lines pooled with both replichores combined.

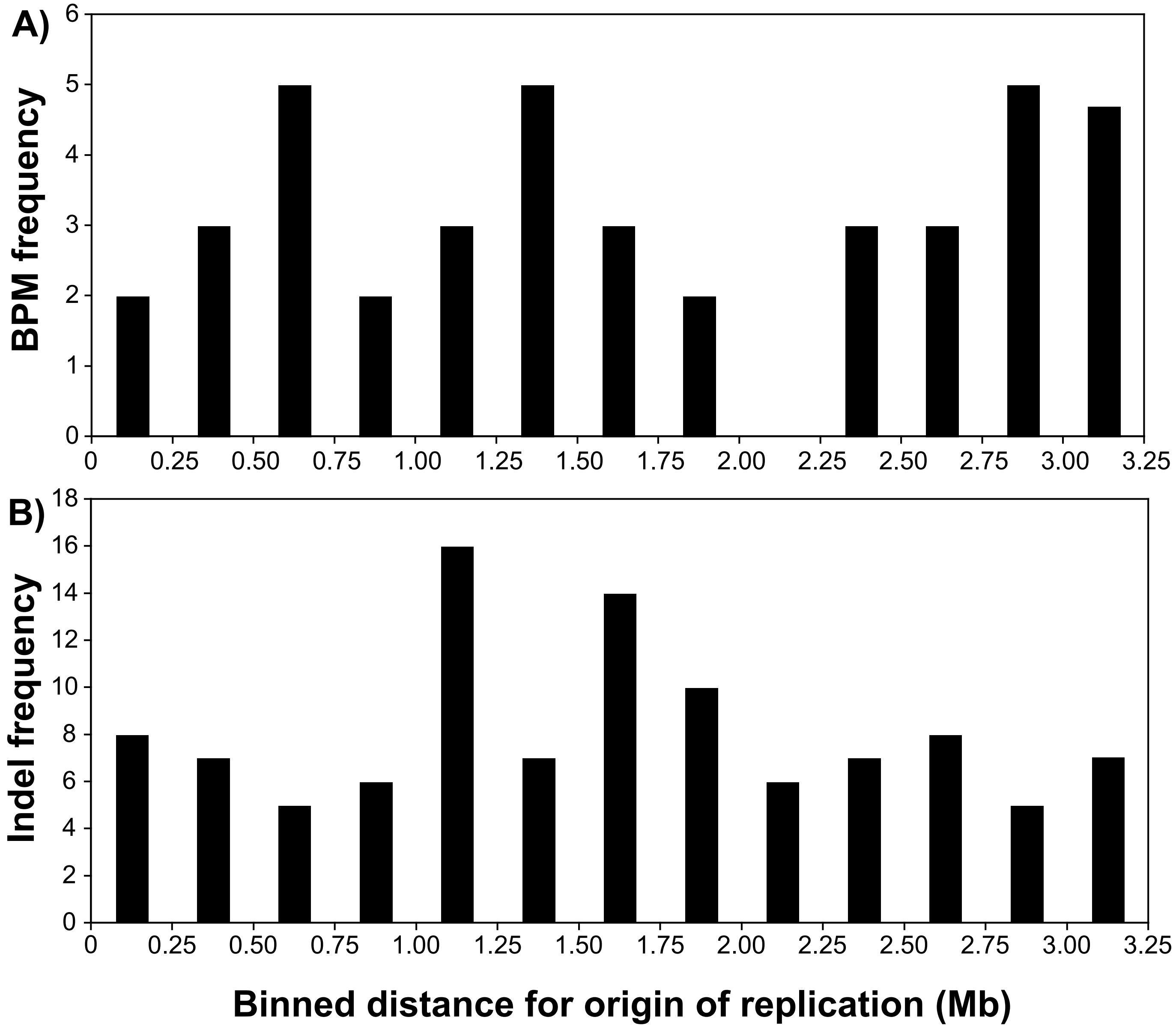

Supplement: Additional file 4: Figure S2. — Frequency of mutations as a function of distance from origin of replication. a) BPMs from wild-type MA lines pooled with both replichores combined. b) Indels from MMR-deficient MA lines pooled with both replichores combined. (PDF 302 kb) [file 12864_2015_2244_MOESM4_ESM.pdf]
