# Supplementary material for: The properties of spontaneous mutations in the opportunistic pathogen Pseudomonas aeruginosa
Source: BMC Genomics. 2016 Jan 5;17:27. doi: 10.1186/s12864-015-2244-3 (PMC4702332; doi:10.1186/s12864-015-2244-3)
Supplement: Additional file 5: Table S3. — Context-dependent mutation rates of focal (center) nucleotides of 64 triplets (top, lagging strand orientation). Rates are per site per generation. (DOC 88 kb) [file 12864_2015_2244_MOESM5_ESM.doc]

**Table S3 Context-dependent mutation rates of focal (center) nucleotides of 64 triplets (top, lagging strand orientation). Rates are per site per generation.**

| **Triplet** | **Right replichore** | **Left replichore** | **Both replichores** |
| --- | --- | --- | --- |
| A**A**A | 9.89 X 10−09 | 0.00 | 4.68 X 10-09 |
| A**A**C | 6.14 X 10−08 | 3.29 X 10−08 | 4.66 X 10-08 |
| A**A**G | 2.83 X 10−09 | 2.11 X 10−08 | 1.23 X 10-08 |
| A**A**T | 8.17 X 10−09 | 7.37 X 10−09 | 7.75 X 10-09 |
| C**A**A | 0.00 | 3.14 X 10−09 | 1.63 X 10-09 |
| C**A**C | 5.09 X 10−08 | 6.60 X 10−08 | 5.86 X 10-08 |
| C**A**G | 3.12 X 10−08 | 3.18 X 10−08 | 3.15 X 10-08 |
| C**A**T | 0.00 | 2.68 X 10−09 | 1.40 X 10-09 |
| G**A**A | 2.23 X 10−09 | 2.06 X 10−09 | 2.14 X 10-09 |
| G**A**C | 4.51 X 10−08 | 3.34 X 10−08 | 3.91 X 10-08 |
| G**A**G | 1.15 X 10−08 | 1.30 X 10−08 | 1.23 X 10-08 |
| G**A**T | 6.48 X 10−09 | 3.96 X 10−09 | 5.17 X 10-09 |
| T**A**A | 0.00 | 0.00 | 0.00 |
| T**A**C | 4.93 X 10−08 | 5.51 X 10−08 | 5.23 X 10-08 |
| T**A**G | 0.00 | 1.38 X 10−08 | 7.12 X 10-09 |
| T**A**T | 0.00 | 0.00 | 0.00 |
| A**C**A | 9.18 X 10−09 | 4.30 X 10−09 | 6.66 X 10-09 |
| A**C**C | 1.27 X 10−08 | 2.20 X 10−08 | 1.75 X 10-08 |
| A**C**G | 1.11 X 10−08 | 1.05 X 10−08 | 1.08 X 10-08 |
| A**C**T | 0.00 | 0.00 | 0.00 |
| C**C**A | 3.27 X 10−09 | 9.18 X 10−09 | 6.32 X 10-09 |
| C**C**C | 2.78 X 10−08 | 3.11 X 10−08 | 2.95 X 10-08 |
| C**C**G | 2.93 X 10−08 | 1.58 X 10−08 | 2.24 X 10-08 |
| C**C**T | 1.66 X 10−09 | 1.56 X 10−09 | 1.61 X 10-09 |
| G**C**A | 1.20 X 10−08 | 9.90 X 10−09 | 1.09 X 10-08 |
| G**C**C | 2.02 X 10−08 | 2.76 X 10−08 | 2.40 X 10-08 |
| G**C**G | 1.75 X 10−08 | 2.07 X 10−08 | 1.91 X 10-08 |
| G**C**T | 8.37 X 10−09 | 6.60 X 10−09 | 7.46 X 10-09 |
| T**C**A | 3.13 X 10−09 | 0.00 | 1.51 X 10-09 |
| T**C**C | 1.07 X 10−08 | 1.99 X 10−09 | 6.18 X 10-09 |
| T**C**G | 9.35 X 10−09 | 1.00 X 10−08 | 9.69 X 10-09 |
| T**C**T | 4.06 X 10−09 | 3.78 X 10−09 | 3.91 X 10-09 |
| A**G**A | 0.00 | 0.00 | 0.00 |
| A**G**C | 1.46 X 10−09 | 0.00 | 7.13 X 10-10 |
| A**G**G | 0.00 | 1.44 X 10−09 | 7.39 X 10-10 |
| A**G**T | 0.00 | 0.00 | 0.00 |
| C**G**A | 3.44 X 10−09 | 3.25 X 10−09 | 3.34 X 10-09 |
| C**G**C | 4.17 X 10−09 | 4.85 X 10−09 | 4.51 X 10-09 |
| C**G**G | 5.22 X 10−09 | 8.37 X 10−09 | 6.83 X 10-09 |
| C**G**T | 5.96 X 10−09 | 3.83 X 10−09 | 4.87 X 10-09 |
| G**G**A | 9.84 X 10−09 | 1.85 X 10−09 | 5.73 X 10-09 |
| G**G**C | 3.56 X 10−09 | 9.60 X 10−09 | 6.64 X 10-09 |
| G**G**G | 8.03 X 10−09 | 5.81 X 10−09 | 6.90 X 10-09 |
| G**G**T | 0.00 | 4.46 X 10−09 | 2.30 X 10-09 |
| T**G**A | 0.00 | 0.00 | 0.00 |
| T**G**C | 0.00 | 3.96 X 10−09 | 2.05 X 10-09 |
| T**G**G | 1.36 X 10−09 | 1.28 X 10−09 | 1.32 X 10-09 |
| T**G**T | 0.00 | 3.63 X 10−09 | 1.89 X 10-09 |
| A**T**A | 0.00 | 0.00 | 0.00 |
| A**T**C | 4.58 X 10−09 | 6.28 X 10−09 | 5.47 X 10-09 |
| A**T**G | 0.00 | 2.48 X 10−09 | 1.29 X 10-09 |
| A**T**T | 0.00 | 6.81 X 10−09 | 3.56 X 10-09 |
| C**T**A | 7.06 X 10−09 | 0.00 | 3.44 X 10-09 |
| C**T**C | 2.41 X 10−09 | 4.57 X 10−09 | 3.52 X 10-09 |
| C**T**G | 9.27 X 10−09 | 7.56 X 10−09 | 8.39 X 10-09 |
| C**T**T | 8.76 X 10−09 | 2.73 X 10−09 | 5.64 X 10-09 |
| G**T**A | 1.94 X 10−08 | 2.67 X 10−08 | 2.32 X 10-08 |
| G**T**C | 7.24 X 10−09 | 1.72 X 10−08 | 1.24 X 10-08 |
| G**T**G | 3.53 X 10−08 | 2.74 X 10−08 | 3.12 X 10-08 |
| G**T**T | 1.88 X 10−08 | 1.53 X 10−08 | 1.70 X 10-08 |
| T**T**A | 0.00 | 0.00 | 0.00 |
| T**T**C | 0.00 | 0.00 | 0.00 |
| T**T**G | 0.00 | 0.00 | 0.00 |
| T**T**T | 0.00 | 0.00 | 0.00 |
