# Supplementary material for: The properties of spontaneous mutations in the opportunistic pathogen Pseudomonas aeruginosa
Source: BMC Genomics. 2016 Jan 5;17:27. doi: 10.1186/s12864-015-2244-3 (PMC4702332; doi:10.1186/s12864-015-2244-3)
Supplement: Additional file 6: Table S4. — Results of model for effect of variables on context-dependent mutation rates of focal nucleotide. (DOC 30 kb) [file 12864_2015_2244_MOESM6_ESM.doc]

**Table S4 Results of model for effect of variables on context-dependent mutation rates of focal nucleotide.**

| **Source** | **DF** | **Sum of Squares** | **F Ratio** | **Prob > F** |
| --- | --- | --- | --- | --- |
| Focal nucleotide (focal) | 3 | 18.66 | 32.16 | <0.0001 |
| 5'-flanking nucleotide (5p) | 3 | 7.87 | 13.56 | <0.0001 |
| 3'-flanking nucleotide (3p) | 3 | 25.17 | 43.39 | <0.0001 |
| focal*5p | 9 | 8.36 | 4.81 | 0.0007 |
| focal*3p | 9 | 39.67 | 22.79 | <0.0001 |
| 5p*3p | 9 | 2.87 | 1.65 | 0.1507 |
